# Supplementary material for: Turning the tide against malaria in high-burden African countries: Trends, threats, and solutions
Source: PLOS Glob Public Health. 2026 May 21;6(5):e0006494. doi: 10.1371/journal.pgph.0006494 (PMC13193415; doi:10.1371/journal.pgph.0006494)
Supplement: S1 Table — (DOCX) [file pgph.0006494.s001.docx]

**S1 Table**. **World Malaria Report data of countries, 2015 – 2024**

|  | **YEARS** | | | | | | | | | |
| --- | --- | --- | --- | --- | --- | --- | --- | --- | --- | --- |
| **Variables** | **2015** | **2016** | **2017** | **2018** | **2019** | **2020** | **2021** | **2022** | **2023** | **2024** |
| **Cases** |  |  |  |  |  |  |  |  |  |  |
| Burkina Faso | 7848131 | 7215226 | 7093584 | 7280725 | 7472680 | 8122685 | 8326915 | 8016213 | 8139355 | 8324000 |
| Cameroon | 5963352 | 6029511 | 5977905 | 6060227 | 5982989 | 6307843 | 6367257 | 6459013 | 7343057 | 7586000 |
| The Democratic Republic of the Congo | 23499389 | 24856401 | 26727855 | 28621752 | 29669385 | 30339645 | 30781802 | 30654224 | 33140568 | 37175000 |
| Ghana | 7819581 | 6776780 | 5914225 | 5226525 | 5150815 | 5227320 | 5245042 | 5315593 | 6551533 | 6740000 |
| Mali | 7097168 | 7184988 | 7469199 | 7710138 | 6863255 | 7586538 | 7744735 | 7988199 | 8229337 | 8475000 |
| Mozambique | 9389988 | 9409618 | 9395822 | 9471126 | 9395404 | 9817892 | 10132564 | 10442873 | 9256415 | 10220000 |
| Niger | 8073585 | 8053568 | 7883346 | 7760340 | 7631511 | 7845383 | 7452978 | 7723787 | 7982516 | 8249000 |
| Nigeria | 54115123 | 55667334 | 57869533 | 59652248 | 61379283 | 65133759 | 65399501 | 66721582 | 68136453 | 68466000 |
| Sudan | 2971363 | 2873064 | 2885551 | 2853785 | 3067521 | 3002954 | 2906886 | 2784704 | 2907170 | 4957000 |
| Tanzania | 7428977 | 7213385 | 6988278 | 6686407 | 6637664 | 7240838 | 7663641 | 7959890 | 8554792 | 9374000 |
| Uganda | 9501063 | 10971082 | 11833607 | 10905744 | 11282360 | 12602807 | 12053928 | 12651126 | 12572518 | 13216000 |
| **Deaths** |  |  |  |  |  |  |  |  |  |  |
| Burkina Faso | 21617 | 18288 | 17297 | 16791 | 16454 | 19176 | 16976 | 16669 | 16146 | 16470 |
| Cameroon | 12200 | 12072 | 11840 | 11679 | 11348 | 13321 | 12783 | 12587 | 11602 | 11590 |
| The Democratic Republic of the Congo | 61075 | 67678 | 69145 | 78205 | 75845 | 75827 | 71017 | 70738 | 67464 | 67710 |
| Ghana | 12800 | 11800 | 11100 | 10700 | 10700 | 10800 | 10800 | 10800 | 11464 | 11590 |
| Mali | 17000 | 14000 | 14600 | 17400 | 17300 | 17200 | 16300 | 15800 | 14203 | 14030 |
| Mozambique | 18300 | 17200 | 15900 | 14900 | 13900 | 14000 | 13500 | 13200 | 17875 | 17690 |
| Niger | 28200 | 29800 | 27100 | 27100 | 27700 | 28400 | 27700 | 27800 | 35381 | 35380 |
| Nigeria | 149000 | 149000 | 151000 | 151000 | 147000 | 155000 | 147000 | 142000 | 184689 | 184830 |
| Sudan | 5050 | 4880 | 4870 | 4550 | 4510 | 4050 | 3810 | 3720 | 6671 | 12810 |
| Tanzania | 22500 | 22500 | 22200 | 22200 | 22400 | 22900 | 23400 | 23500 | 25540 | 26230 |
| Uganda | 14700 | 14400 | 14400 | 13800 | 14200 | 14200 | 13900 | 13900 | 13800 | 16470 |

**Note**. The latest adjusted case and death data (2015 – 2022) were extracted from the World Malaria Report 2023. The data for the years 2023 and 2024 were extracted from the World Malaria Reports 2024 and 2025, respectively.
