# Supplementary material for: Turning the tide against malaria in high-burden African countries: Trends, threats, and solutions
Source: PLOS Glob Public Health. 2026 May 21;6(5):e0006494. doi: 10.1371/journal.pgph.0006494 (PMC13193415; doi:10.1371/journal.pgph.0006494)
Supplement: S2 Table — (DOCX) [file pgph.0006494.s002.docx]

**S2 Table. PubMed-based search strategies used to identify relevant papers on the status of WHO biological threats to malaria control in HBHI countries.**

| **Categories** | **Search strategies** |
| --- | --- |
| Antimalarial drug resistance | (“Plasmodium falciparum”[MeSH Terms] OR “Plasmodium falciparum”[tiab]) AND (“kelch13”[tiab] OR “K13”[tiab] OR “kelch propeller domain”[tiab]) AND (“artemisinin resistance”[tiab] OR “drug resistance, malaria”[MeSH Terms] OR “antimalarial resistance”[tiab]) AND (“epidemiology”[Subheading] OR “epidemiology”[tiab] OR “prevalence”[tiab] OR “distribution”[tiab] OR “molecular surveillance”[tiab]) AND (“Africa South of the Sahara”[MeSH Terms] OR Africa[tiab] OR “Burkina Faso”[tiab] OR Cameroon[tiab] OR “Democratic Republic of the Congo”[tiab] OR DRC[tiab] OR Ghana[tiab] OR Mali[tiab] OR Mozambique[tiab] OR Niger[tiab] OR Nigeria[tiab] OR Sudan[tiab] OR Tanzania[tiab] OR Uganda[tiab]). |
| Antimalarial drug efficacy | (“Artemisinin-Based Combination Therapies”[MeSH Terms] OR “ACT”[tiab] OR “artemisinin combination therapy”[tiab] OR “artemether-lumefantrine”[tiab] OR “dihydroartemisinin-piperaquine”[tiab]) AND (“treatment outcome”[MeSH Terms] OR “treatment efficacy”[tiab] OR “clinical efficacy”[tiab] OR “therapeutic efficacy”[tiab] OR “treatment failure”[tiab]) AND (“Plasmodium falciparum”[MeSH Terms] OR “falciparum malaria”[tiab]) AND (“Africa South of the Sahara”[MeSH Terms] OR Africa[tiab] OR “Burkina Faso”[tiab] OR Cameroon[tiab] OR “Democratic Republic of the Congo”[tiab] OR DRC[tiab] OR Ghana[tiab] OR Mali[tiab] OR Mozambique[tiab] OR Niger[tiab] OR Nigeria[tiab] OR Sudan[tiab] OR Tanzania[tiab] OR Uganda[tiab]) |
| *Anopheles* insecticide resistance | (“Anopheles”[MeSH Terms] OR Anopheles[tiab]) AND (“insecticide resistance”[MeSH Terms] OR “insecticide resistance”[tiab] OR “vector resistance”[tiab]) AND (carbamates[tiab] OR neonicotinoids[tiab] OR organochlorines[tiab] OR organophosphates[tiab] OR pyrethroids[tiab] OR pyrroles[tiab]) AND (“epidemiology”[Subheading] OR “distribution”[tiab] OR “prevalence”[tiab] OR “surveillance”[tiab]) AND (“Africa South of the Sahara”[MeSH Terms] OR Africa[tiab] OR “Burkina Faso”[tiab] OR Cameroon[tiab] OR “Democratic Republic of the Congo”[tiab] OR DRC[tiab] OR Ghana[tiab] OR Mali[tiab] OR Mozambique[tiab] OR Niger[tiab] OR Nigeria[tiab] OR Sudan[tiab] OR Tanzania[tiab] OR Uganda[tiab]) |
| *P. falciparum* histidine-rich protein 2 gene deletions | (“Plasmodium falciparum”[MeSH Terms] OR “Plasmodium falciparum”[tiab]) AND (“histidine-rich protein 2”[tiab] OR “HRP2”[tiab] OR “pfhrp2 deletion”[tiab] OR “hrp2 gene deletion”[tiab]) AND (“gene deletion”[MeSH Terms] OR deletion*[tiab]) AND (“epidemiology”[Subheading] OR “prevalence”[tiab] OR “distribution”[tiab] OR “molecular surveillance”[tiab]) AND (“Africa South of the Sahara”[MeSH Terms] OR Africa[tiab] OR “Burkina Faso”[tiab] OR Cameroon[tiab] OR “Democratic Republic of the Congo”[tiab] OR DRC[tiab] OR Ghana[tiab] OR Mali[tiab] OR Mozambique[tiab] OR Niger[tiab] OR Nigeria[tiab] OR Sudan[tiab] OR Tanzania[tiab] OR Uganda[tiab]) |
| *Anopheles stephensi* invasion | (“Anopheles stephensi”[MeSH Terms] OR “Anopheles stephensi”[tiab]) AND (“invasive species”[MeSH Terms] OR invasion[tiab] OR “range expansion”[tiab] OR “geographic spread”[tiab] OR introduction[tiab]) AND (“vector distribution”[tiab] OR “vector surveillance”[tiab] OR “entomological surveillance”[tiab]) AND (“Africa South of the Sahara”[MeSH Terms] OR Africa[tiab] OR “Burkina Faso”[tiab] OR Cameroon[tiab] OR “Democratic Republic of the Congo”[tiab] OR DRC[tiab] OR Ghana[tiab] OR Mali[tiab] OR Mozambique[tiab] OR Niger[tiab] OR Nigeria[tiab] OR Sudan[tiab] OR Tanzania[tiab] OR Uganda[tiab]) |
