# Supplementary figures and images for: Turning the tide against malaria in high-burden African countries: Trends, threats, and solutions
Source: PLOS Glob Public Health. 2026 May 21;6(5):e0006494. doi: 10.1371/journal.pgph.0006494 (PMC13193415; doi:10.1371/journal.pgph.0006494)

**S1 Fig**. **Mann–Kendall trend analysis of cases of countries, 2015 – 2024.**


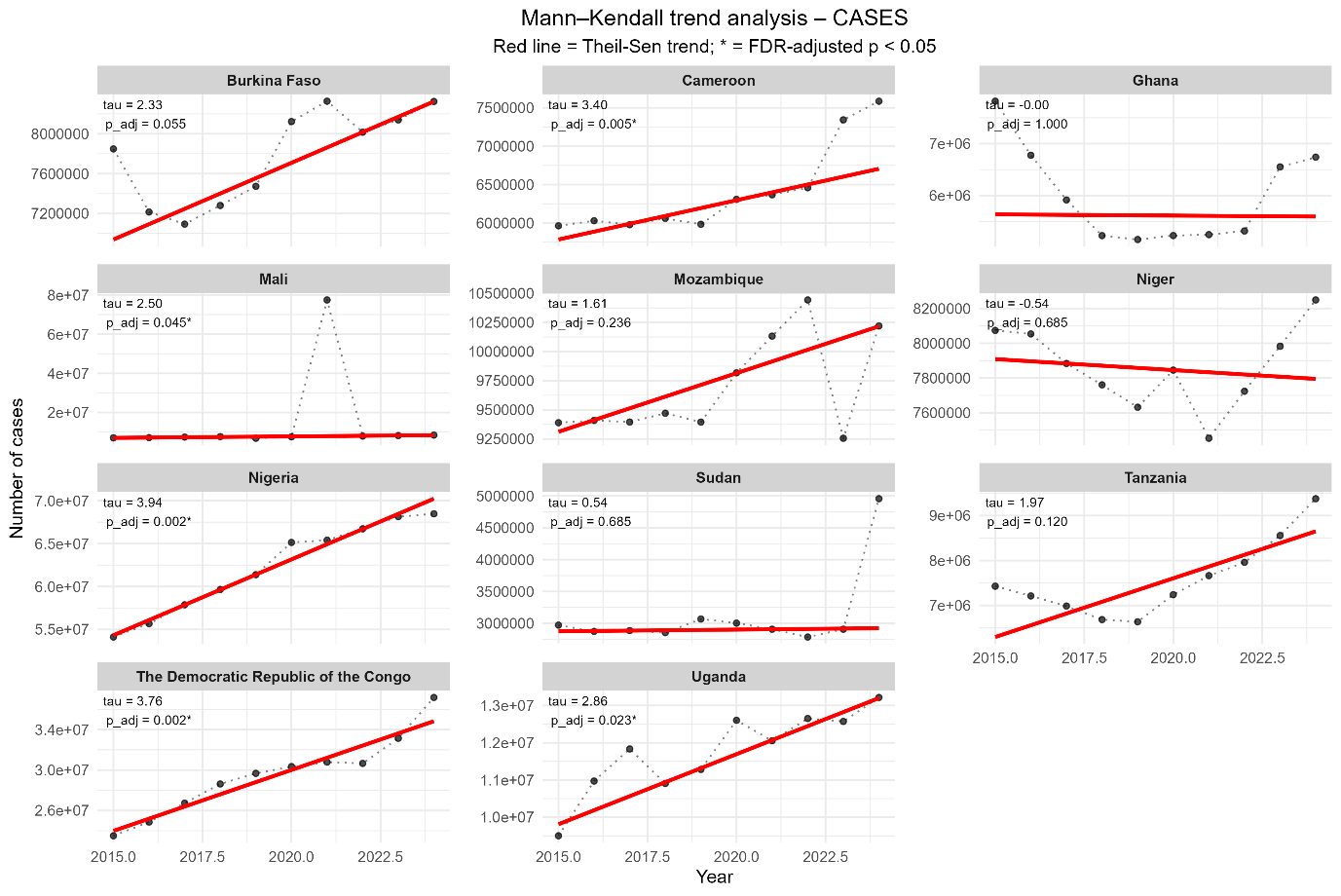

Supplement: S1 Fig — (DOCX) [file pgph.0006494.s003.docx]

**S2 Fig**. **Mann–Kendall trend analysis of deaths of countries, 2015 – 2024.**


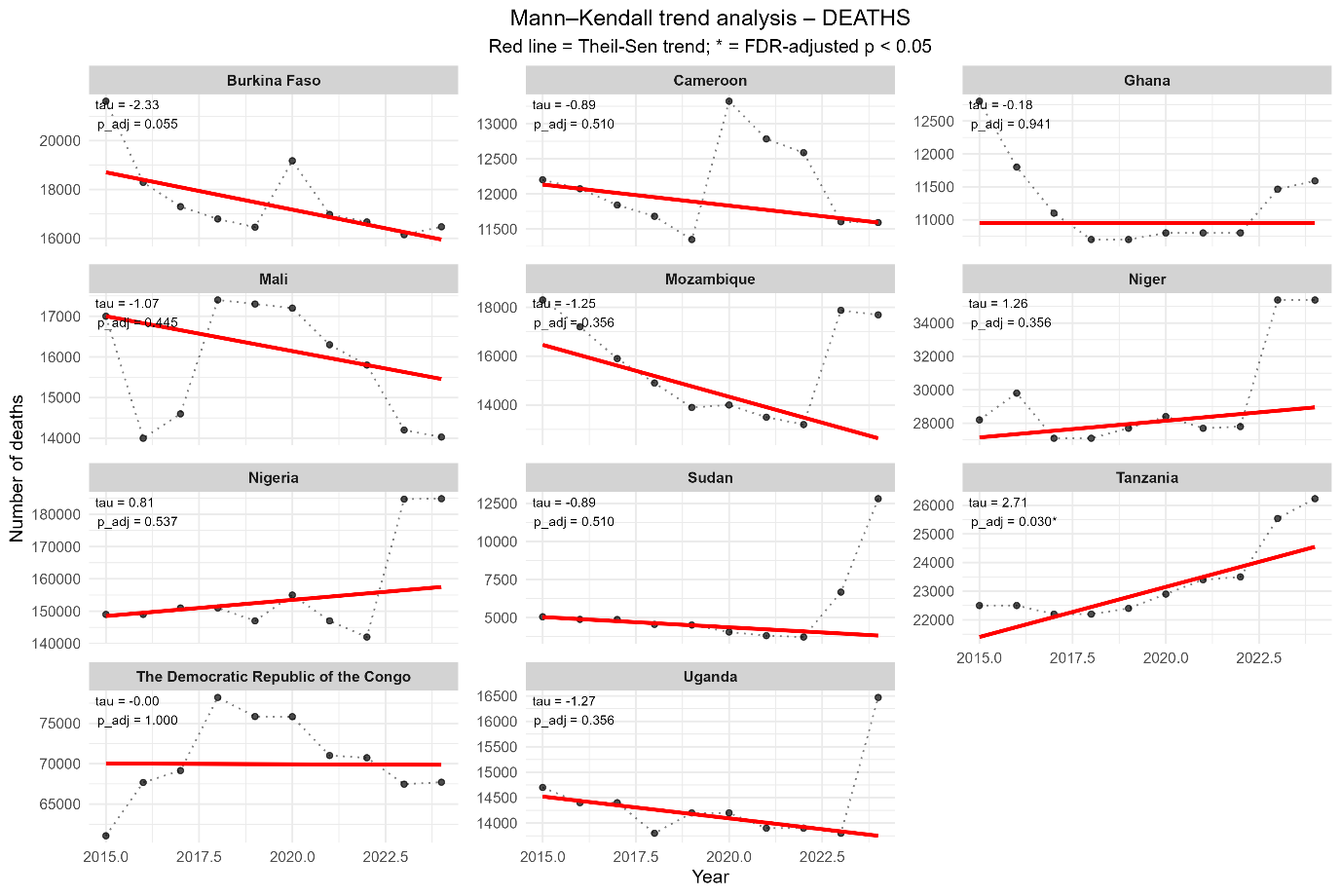

Supplement: S2 Fig — (DOCX) [file pgph.0006494.s004.docx]
