# Supplementary material for: Turning the tide against malaria in high-burden African countries: Trends, threats, and solutions
Source: PLOS Glob Public Health. 2026 May 21;6(5):e0006494. doi: 10.1371/journal.pgph.0006494 (PMC13193415; doi:10.1371/journal.pgph.0006494)
Supplement: Attachment — Submitted filename: PGPH-D-26-00803_Anonymous_Reviewer_Comments.docx [file pgph.0006494.s005.docx]

**Turning the tide against malaria in high-burden African countries: Trends, threats, and solutions**

**Major comments**

1. The manuscript is presented as a Research Article, but much of the content reads as a narrative review or policy commentary supported by a limited descriptive secondary analysis. The authors should either substantially strengthen the analytical component or reconsider the framing of the manuscript. This concern is evident in the abstract and methods, especially around lines 31–37 and 108–156.
2. The abstract and methods indicate that the analysis covers 2015–2024 (line 31; line 119), but Figure 2 is labelled 2015–2023 (line 199) and Figure 3 is also labelled 2015–2023 (line 212). At the same time, the Results section discusses specific 2024 values and 2024 changes. The study period must be made fully consistent across abstract, methods, results, figures, tables, and references.
3. The Results section states that Nigeria had approximately 8 million cases in 2015 and 67 million in 2024 (line 173), whereas the Figure 2 caption states that Nigeria rose from about 8 million in 2015 to 24 million in 2023 (line 204). In addition, the stated “32.40-fold increase” does not appear consistent with the numbers reported. These discrepancies substantially weaken confidence in the dataset and analysis and should be corrected throughout. Relevant lines include 173–180 and 204–208.
4. At line 198, the manuscript refers to Figure 3 in support of statements about increases in malaria cases, but Figure 3 is described as a figure about mortality beginning at line 212. Similarly, at line 194, the manuscript states that “MK analysis also revealed a decrease in malaria cases” in Uganda, although the preceding discussion is focused on mortality trends (lines 185–193). The authors should carefully revise the Results so that cases and deaths are not interchanged.
5. The manuscript indicates that WHO reports, the WHO Malaria Threats Map, and a structured literature review were used, but the methods do not provide enough detail on data extraction, handling of report years versus data years, literature search strategy, study selection, or integration of narrative evidence with the quantitative analysis. The choice of the Mann–Kendall test is reasonable for monotonic trend assessment, but the methodological description is currently too limited for the scope of the conclusions drawn. Please revise lines 119–156 accordingly.
6. The manuscript makes broad claims regarding health-system weakness, workforce limitations, underuse of diagnostics, donor dependence, corruption, climate change, armed conflict, and population movement. Many of these are plausible and important, but the paper should more clearly distinguish between what is directly demonstrated by the data, what is inferred from the literature, and what is being proposed as a strategic recommendation. At present, the conclusions are broader than the quantitative analysis alone can justify. This applies particularly to lines 342–467.
7. The manuscript compares countries mainly using absolute malaria case and death counts, even though the included countries differ substantially in population size, epidemiological context, and reporting systems. The analysis would be strengthened by incorporating standardized measures such as incidence and mortality rates, and by clearly stating whether the values used are reported cases, WHO estimates, or another metric. This concern affects the interpretation of lines 157–198 and Figures 1–3.
8. The submission indicates “Data Availability: Not applicable.” For a manuscript based on extracted and harmonized public data, this is not sufficient. The extracted dataset underlying the analyses, and ideally the code used for trend testing and figure generation, should be made available. This is especially important given the numerical inconsistencies noted above.
9. The manuscript text states that primaquine is recommended for radical cure of *P. vivax* in Tanzania and Sudan (line 240), but Table 2 appears to mark this as “n/a,” including for Sudan/Tanzania. In addition, the notation “–3” is unclear and non-standard; the footnote reportedly explains “3,” but not the prefixed dash/minus sign. Table 2 should be revised for clarity, consistency, and direct alignment with the narrative text.
10. The authors should verify that each citation supports the exact claim being made at sentence level. In addition, the reference list appears to contain formatting/export errors that should be corrected before further consideration.

**Minor comments**

1. Please check arithmetic and wording in the Results section, especially around lines 178–183, and correct the likely typographical error “204” where 2024 is intended.
2. Some figure captions are more interpretive than descriptive. The legends for Figures 1–5 would benefit from being more concise and factual, with interpretation reserved for the main text.
3. Terms used in the tables, including “mixed,” “n/a,” and implementation categories, should be clearly defined in the legends or footnotes.
4. The manuscript would benefit from a full technical and language edit to improve consistency, terminology, and scientific tone.

Overall, I find the topic important and potentially suitable for the journal, but the manuscript in its current form has substantial issues that need to be addressed through major revision before it can be evaluated further.
