# Supplementary material for: Turning the tide against malaria in high-burden African countries: Trends, threats, and solutions
Source: PLOS Glob Public Health. 2026 May 21;6(5):e0006494. doi: 10.1371/journal.pgph.0006494 (PMC13193415; doi:10.1371/journal.pgph.0006494)
Supplement: Attachment — Submitted filename: Response to Reviewers_R1.docx [file pgph.0006494.s007.docx]

**Manuscript** ID: PGPH-D-26-00803

**Title**: Turning the tide against malaria in high-burden African countries: Trends, threats, and solutions

**Journal**: PLOS Global Public Health

Dear Editors-in-Chief, Dr Catherine Kyobutungi and Dr Manuela De Allegri

We thank you for the opportunity to revise and resubmit our manuscript.

In this letter, we provide a detailed, point-by-point response to all comments raised by the Editor and the Reviewers. We are grateful for the reviewers’ thoughtful and constructive feedback, which we believe has significantly improved the quality and clarity of our manuscript.

Please find below our responses to each of the reviewers’ comments (indicated in brown). All corresponding revisions have been incorporated into the revised manuscript and are highlighted in yellow for ease of reference. We confirm that all requested revisions have been carefully addressed.

We hope that the revised version meets the journal’s standards and look forward to your favourable consideration.

Sincerely,

**Loick P. Kojom Foko**

CEREBA, Douala-Cameroon

The University of Douala-Cameroon

ICGEB, New Delhi-India

Email: [kojomloick@gmail.com](mailto:kojomloick@gmail.com)

**Journal Requirements:**

1. Please provide a complete Data Availability Statement in the submission form, ensuring you include all necessary access information or a reason for why you are unable to make your data freely accessible. If your research concerns only data provided within your submission, please write "All data are in the manuscript and/or supporting information files" as your Data Availability Statement. For further assistance, you may go to: <http://journals.plos.org/globalpublichealth/s/data-availability>

**Reply**: Corrected.

2. Please provide separate main figure files in .tif or .eps format only and ensure that all files are under our size limit of 10MB. For more information about how to convert your figure files please see our guidelines: https://journals.plos.org/globalpublichealth/s/figures

**Reply**: Done.

3. Some material included in your submission may be copyrighted. According to PLOS’s copyright policy, authors who use figures or other material (e.g., graphics, clipart, maps) from another author or copyright holder must demonstrate or obtain permission to publish this material under the Creative Commons Attribution 4.0 International (CC BY 4.0) License used by PLOS journals. Please closely review the details of PLOS’s copyright requirements here: PLOS Licenses and Copyright. If you need to request permissions from a copyright holder, you may use PLOS's Copyright Content Permission form.

**Reply**: We have provided details in Figure 6.

**Reviewer #1:** This manuscript addresses an important and timely public health question and has potential value as a broad synthesis of malaria trends, threats, and strategic responses in high-burden African countries. The topic is clearly relevant to the readership of PLOS Global Public Health. However, in its current form, the manuscript requires major revision before it can be considered further. My main concerns relate to article framing, internal consistency of the results, adequacy of methodological detail, interpretation of the findings, data availability, and consistency between the text, figures, and tables.

**Major comments**

1. The manuscript is presented as a Research Article, but much of the content reads as a narrative review or policy commentary supported by a limited descriptive secondary analysis. The authors should either substantially strengthen the analytical component or reconsider the framing of the manuscript. This concern is evident in the abstract and methods, especially around lines 31–37 and 108–156.

**Reply**: We have revised the manuscript as proposed. The analytical component of the paper has been strengthened by adding details in the main text and supporting information on search strategies for WHO Biological Threat sections. We have also improved the statistical analysis section (MK analysis). We have added a figure to depict the MK analysis, and details have been presented in Supplementary files 3 and 4. The revisions are as follows:

**Data sources**

Country-level malaria data were obtained from publicly available World Health Organization (WHO) sources. Primary epidemiological data for the 11 HBHI countries were extracted from successive editions of the World Malaria Report (2016–2025), which provide standardized annual estimates of malaria cases and deaths. When inspecting malaria burden data from World Malaria Reports (WMR) 2016 to 2025, we have observed discrepancies in the same data from one report to another, which are due to WHO-made adjustments. Thus, for extracted data for the period 2015-2022, we have used the WMR 2023 data (**S1 Table**). For the data of years 2023 and 2024, the WMR 2024 and 2025 were used, respectively (**S1 Table**). Data were collated for the period 2015–2024 to enable consistent trend analysis across countries.

Information on national malaria control strategies implemented during the study period was abstracted from WHO reports and country profiles. Data on the current status of biological threats to malaria control and elimination—including antimalarial drug resistance, insecticide resistance, and diagnostic challenges—were retrieved from the WHO Malaria Threats Map (<https://apps.who.int/malaria/maps/threats/>). Where multiple updates were available, the most recent information within the study period was used, and data were aligned to the corresponding country-year as far as possible.

To contextualize WHO biological threats to malaria control in HBHI countries, i.e., antimalarial drug resistance and efficacy, *P. falciparum* histidine-rich protein 2 gene (*pfhrp2*) deletions, vector insecticide resistance, and *Anopheles stephensi* invasion, and validate reported patterns, we conducted a structured literature review using PubMed. The search was restricted to English-language peer-reviewed articles and technical reports published between 2015 and 2024. Title and abstract screening were conducted to assess relevance to malaria control interventions and resistance patterns, followed by full-text review of eligible records. Searches were supplemented through expert consultation and backward reference mining of relevant publications. Only peer-reviewed articles and authoritative technical reports were included for critical appraisal and synthesis. The details of each search strategy, tailored to each WHO biological threat, are presented in **S2 Table**. For instance, the search strategy used for *pfhrp2* deletions is delineated as follows: (“Plasmodium falciparum”[MeSH Terms] OR “Plasmodium falciparum”[tiab]) AND (“histidine-rich protein 2”[tiab] OR “HRP2”[tiab] OR “pfhrp2 deletion”[tiab] OR “hrp2 gene deletion”[tiab]) AND (“gene deletion”[MeSH Terms] OR deletion*[tiab]) AND (“epidemiology”[Subheading] OR “prevalence”[tiab] OR “distribution”[tiab] OR “molecular surveillance”[tiab]) AND (“Africa South of the Sahara”[MeSH Terms] OR Africa[tiab] OR “Burkina Faso”[tiab] OR Cameroon[tiab] OR “Democratic Republic of the Congo”[tiab] OR DRC[tiab] OR Ghana[tiab] OR Mali[tiab] OR Mozambique[tiab] OR Niger[tiab] OR Nigeria[tiab] OR Sudan[tiab] OR Tanzania[tiab] OR Uganda[tiab]). No formal quality appraisal or meta-analysis was conducted, consistent with the narrative and contextual nature of the review.

**Data management and analysis**

Extracted data were compiled into a harmonized database and cross-checked for internal consistency. Descriptive statistics were used to summarize annual malaria cases, deaths, and reported biological threats across countries and over time. Data visualization was conducted using GraphPad Prism v8.02 (GraphPad Software, CA, USA) to illustrate temporal trajectories and comparative patterns.

Temporal trends in annual malaria cases and deaths from 2015 to 2024 were assessed separately for each of the 11 HBHI countries using the Mann–Kendall (MK) trend test. The MK test is a non-parametric method appropriate for detecting monotonic trends in time-series data and is robust to non-normality and heteroscedasticity, which are common in surveillance datasets. Annual country-level counts of malaria cases and deaths were extracted and structured as univariate time series, with each observation corresponding to a single year. Analyses were conducted on available data without imputation; countries with incomplete time series were analyzed using all observed data points. The MK test assesses the presence of a monotonic trend by evaluating the relative ranking of observations over time and computing Kendall’s tau (τ), which quantifies both the direction and strength of the trend. Thus, a positive τ value indicates an increasing trend, whereas a negative τ value indicates a decreasing trend. Sen Slope corrections for autocorrelation were applied. The Theil–Sen slope method was used to estimate the slope (trend). This method is widely used to compute trends because it provides a robust and reliable estimate of slope that is not unduly influenced by outliers or non-normal data distributions [5]. Analyses were performed in R version 4.5 using the “trend” package. The mk.test() function was applied to each country-specific time series, with variance correction automatically implemented to account for tied values where present. Statistical significance was defined a priori as a two-sided *p*-value < 0.05. No adjustment for multiple comparisons was performed, as analyses were conducted independently for each country.

2. The abstract and methods indicate that the analysis covers 2015–2024 (line 31; line 119), but Figure 2 is labelled 2015–2023 (line 199) and Figure 3 is also labelled 2015–2023 (line 212). At the same time, the Results section discusses specific 2024 values and 2024 changes. The study period must be made fully consistent across abstract, methods, results, figures, tables, and references.

**Reply**: We have addressed these typos in the revised paper.

3. The Results section states that Nigeria had approximately 8 million cases in 2015 and 67 million in 2024 (line 173), whereas the Figure 2 caption states that Nigeria rose from about 8 million in 2015 to 24 million in 2023 (line 204). In addition, the stated “32.40-fold increase” does not appear consistent with the numbers reported. These discrepancies substantially weaken confidence in the dataset and analysis and should be corrected throughout. Relevant lines include 173–180 and 204–208.

**Reply**: We have adjusted this mistake as follows here. Also, we have added the data from World Malaria Reports 2023, 2024, and 2025, used to perform analyses.

Overall, morbidity cases have been on a constant rise from 2015 to 2024 for several countries, including Cameroon, DRC, Nigeria, and Uganda. Nigeria reported ~54.1 million cases in 2015 and ~68.5 million in 2024. In Cameroon, morbidity cases jumped from ~.9 million in 2015 to ~7.6 million in 2024, whereas in the DRC the cases increased from ~23.5 million in 2015 to ~37.2 million in 2024.

4. At line 198, the manuscript refers to Figure 3 in support of statements about increases in malaria cases, but Figure 3 is described as a figure about mortality beginning at line 212. Similarly, at line 194, the manuscript states that “MK analysis also revealed a decrease in malaria cases” in Uganda, although the preceding discussion is focused on mortality trends (lines 185–193). The authors should carefully revise the Results so that cases and deaths are not interchanged.

**Reply**: We have revised the text and structured it to first address morbidity, and then mortality.

The malaria case trends during 2015 – 2024 for each HBHI country reveal contrasting patterns (**Fig 2**). Overall, morbidity cases have been on a constant rise from 2015 to 2024 for several countries, including Cameroon, DRC, Nigeria, and Uganda. Nigeria reported ~54.1 million cases in 2015 and ~68.5 million in 2024. In Cameroon, morbidity cases jumped from ~.9 million in 2015 to ~7.6 million in 2024, whereas in the DRC the cases increased from ~23.5 million in 2015 to ~37.2 million in 2024. This increase in malaria cases was even observed before the COVID-19 pandemic in 2020 in countries such as Burkina Faso, Cameroon, Uganda, and Nigeria (**Fig 2**). The highest increments in morbidity were observed in Sudan in 2024 (1.71-fold increase), Ghana in 2023 (1.23-fold increase), Uganda in 2016 (1.15-fold increase) and 2020 (1.12-fold increase), Cameroon in 2023 (1.14-fold increase), DRC in 2024 (1.12-fold increase), and Mali in 2020 (1.11-fold increase) (**Fig 3**). These observations were confirmed by the MK analyses. Indeed, five countries witnessed statistically significant increases in malaria across the years, namely Nigeria (MK statistics = 3.94, *p* = 0.002), DRC (MK statistics = 3.76, *p* = 0.002), Cameroon (MK statistics = 3.40, *p* = 0.005), Uganda (MK statistics = 2.86, *p* = 0.02), and Mali (MK statistics = 2.50, *p* = 0.04). For Burkina Faso, there was an increase in malaria cases, but the statistical significance was borderline (MK statistics = 2.33, *p* = 0.055) (**Fig 4 & S3 Fig**).

Regarding deaths, the temporal patterns have varied between countries, with four patterns: i) gradual increase followed by gradual decrease (inverted U-like) (DRC), ii) relative stagnation followed by an increase (Uganda, Tanzania, Niger, Nigeria, and Sudan), iii) gradual decrease followed by gradual increase (U-like) (Ghana, Mozambique), and jagged-like (Burkina Faso, Cameroon, Mali) (**Fig 2**). For instance, in Ghana, the number of deaths dropped from 12,800 in 2015 to 10,800 in 2022, then increased to 11,590 in 2024. In Nigeria, the number of deaths increased from 149,000 in 2015 to 184,830 in 2024 (**Fig 2**). The highest increments in death cases were observed in Sudan in 2023 (1.79-fold increase) and 2024 (1.92-fold increase) compared to years 2022 and 2023, respectively (**Fig 5**). The MK analysis revealed a statistically significant increase in death cases in Tanzania (MK statistics = 2.71, *p* = 0.03) (**Fig 4 & S4 Fig**). The remaining countries have not experienced a statistically significant decrease or increase in malaria deaths (**Fig 4 & S3 Fig**).

5. The manuscript indicates that WHO reports, the WHO Malaria Threats Map, and a structured literature review were used, but the methods do not provide enough detail on data extraction, handling of report years versus data years, literature search strategy, study selection, or integration of narrative evidence with the quantitative analysis. The choice of the Mann–Kendall test is reasonable for monotonic trend assessment, but the methodological description is currently too limited for the scope of the conclusions drawn. Please revise lines 119–156 accordingly.

**Reply**: We have addressed this point in the reply to comment number 1.

6. The manuscript makes broad claims regarding health-system weakness, workforce limitations, underuse of diagnostics, donor dependence, corruption, climate change, armed conflict, and population movement. Many of these are plausible and important, but the paper should more clearly distinguish between what is directly demonstrated by the data, what is inferred from the literature, and what is being proposed as a strategic recommendation. At present, the conclusions are broader than the quantitative analysis alone can justify. This applies particularly to lines 342–467.

**Reply**: We have added a paragraph to address this important point. Also, the conclusion section has been revised as below.

A priority strategic recommendation emerging from this work is the strengthening of integrated, real-time malaria surveillance and response systems at the health-facility level. Given the demonstrated increase in malaria morbidity in several HBHI countries (e.g., Nigeria, DRC, Cameroon, Mali, and Uganda) and the heterogeneity of trends across settings, timely and granular data are essential to guide adaptive interventions. This could involve the deployment of digital reporting platforms (i.e., malaria dashboards) that enable near-real-time case notification, integration of routine diagnostic data, and systematic monitoring of WHO biological threats to malaria control. Such strategies should be coupled with decentralized decision-making capacity, allowing district-level health teams to rapidly adjust control strategies (e.g., targeted vector control). Although not directly assessed in this analysis, evidence from prior implementations suggests that strengthened surveillance systems can improve responsiveness and efficiency of malaria control programs, making them a critical component for accelerating progress toward elimination in HBHI settings.

**Concluding remarks**

Here, we analyzed the epidemiological trends of the malaria burden in HBHI countries, identified knowledge and practice gaps, and proposed tailored solutions to control malaria in the upcoming years. The quantitative analysis demonstrates that malaria morbidity has increased significantly in five HBHI countries (Nigeria, DRC, Cameroon, Mali, and Uganda) between 2015 and 2024, while mortality trends remain heterogeneous and mostly non-significant across settings. Due to these observed rises in malaria morbidity in these HBHI countries and the varying trends across different settings, it is crucial to produce timely and detailed data for guiding adaptive interventions. Strengthened surveillance systems should be implemented to enhance responsiveness and efficiency in malaria control efforts.

7. The manuscript compares countries mainly using absolute malaria case and death counts, even though the included countries differ substantially in population size, epidemiological context, and reporting systems. The analysis would be strengthened by incorporating standardized measures such as incidence and mortality rates, and by clearly stating whether the values used are reported cases, WHO estimates, or another metric. This concern affects the interpretation of lines 157–198 and Figures 1–3.

**Reply**: We have compared the countries in terms of fold changes, not in terms of counts. In general, when presenting counts, the fold changes are also associated with each country. An analysis based on fold changes intrinsically takes into account differences in the differences of countries between population size. Also, our MK analyses were performed for each country by using their own data. Note that the WHO use the data provided by the National Malaria Control Programme of each country, which varies as per reporting systems. Thus, the diagnostic bias outlined by the reviewer is also introduced in WHO assessments. Thus, we have added a limitation section below as:

**Limitations**

A key limitation of this study is that this prospective analysis is based on absolute malaria case and death counts, which may be influenced by external factors not explicitly accounted for. Changes in population size and demographic structure across countries over the study period could affect the observed trends, as increases in cases may partly reflect population growth rather than true changes in transmission intensity. In addition, temporal improvements in surveillance systems, reporting completeness, and the adoption of more sensitive diagnostic may have contributed to apparent increases or fluctuations in reported cases and deaths. These factors should be considered when interpreting the observed temporal trends. Despite these considerations, the results still offer useful insights into multi-year temporal trends in malaria burden across diverse epidemiological settings.

8. The submission indicates “Data Availability: Not applicable.” For a manuscript based on extracted and harmonized public data, this is not sufficient. The extracted dataset underlying the analyses, and ideally the code used for trend testing and figure generation, should be made available. This is especially important given the numerical inconsistencies noted above.

**Reply**: Corrected.

9. The manuscript text states that primaquine is recommended for radical cure of *P. vivax* in Tanzania and Sudan (line 240), but Table 2 appears to mark this as “n/a,” including for Sudan/Tanzania. In addition, the notation “–3” is unclear and non-standard; the footnote reportedly explains “3,” but not the prefixed dash/minus sign. Table 2 should be revised for clarity, consistency, and direct alignment with the narrative text.

**Reply**: We have mentioned Zanzibar (in Tanzania). In the table, Tanzania is split into Zanzibar and the Mainland. We have adjusted the notation as suggested. The table depicts the strategies used in these countries to control malaria. It is in line with the ‘Key malaria control strategies adopted by HBHI countries’ section.

10. The authors should verify that each citation supports the exact claim being made at sentence level. In addition, the reference list appears to contain formatting/export errors that should be corrected before further consideration.

**Reply**: We have addressed the references with formatting/export errors. Thank you.

**Minor comments**

1. Please check arithmetic and wording in the Results section, especially around lines 178–183, and correct the likely typographical error “204” where 2024 is intended.

**Reply**: Corrected.

2. Some figure captions are more interpretive than descriptive. The legends for Figures 1–5 would benefit from being more concise and factual, with interpretation reserved for the main text.

**Reply**: Corrected.

3. Terms used in the tables, including “mixed,” “n/a,” and implementation categories, should be clearly defined in the legends or footnotes.

**Reply**: Corrected.

4. The manuscript would benefit from a full technical and language edit to improve consistency, terminology, and scientific tone.

**Reply**: Corrected.

Overall, I find the topic important and potentially suitable for the journal, but the manuscript in its current form has substantial issues that need to be addressed through major revision before it can be evaluated further.

**Reply**: Thank you for this constructive analysis.

**Reviewer #2**: The manuscript provides a timely and critical analysis of malaria epidemiology.

The article does not discuss confounders of absolute numbers like population growth or changes in notification and surveillance data and diagnostic tools. Neither does it recognize the small sample size as a limitation.

**Reply**: We have added a limitation section as below:

**Limitations**

A key limitation of this study is that this prospective analysis is based on absolute malaria case and death counts, which may be influenced by external factors not explicitly accounted for. Changes in population size and demographic structure across countries over the study period could affect the observed trends, as increases in cases may partly reflect population growth rather than true changes in transmission intensity. In addition, temporal improvements in surveillance systems, reporting completeness, and the adoption of more sensitive diagnostic may have contributed to apparent increases or fluctuations in reported cases and deaths. These factors should be considered when interpreting the observed temporal trends. Despite these considerations, the results still offer useful insights into multi-year temporal trends in malaria burden across diverse epidemiological settings.

The authors should also provide the secondary analysis of the WHO data as a supplementary file.

**Reply**: Done (Supplementary file 1).
